# Exposure to Hedione Increases Reciprocity in Humans

**DOI:** 10.3389/fnbeh.2017.00079

**Published:** 2017-05-02

**Authors:** Sebastian Berger, Hanns Hatt, Axel Ockenfels

**Affiliations:** ^1^Department of Organization, Department of Organization and Human Resource Management, University of BernBern, Switzerland; ^2^Department of Cell Physiology, Ruhr-University BochumBochum, Germany; ^3^Department of Economics, University of CologneCologne, Germany

**Keywords:** hedione, chemosignals, cooperation, reciprocity, altruistic punishment, experimental games

## Abstract

Cooperation among unrelated humans is frequently regarded as a defining feature in the evolutionary success of our species. Whereas, much research has addressed the strategic and cognitive mechanisms that underlie cooperation, investigations into chemosensory processes have received very limited research attention. To bridge that gap, we build on recent research that has identified the chemically synthesized odorant Hedione (HED) as a ligand for the putative human pheromone receptor (VN1R1) expressed in the olfactory mucosa, and hypothesize that exposure to HED may increase reciprocity. Applying behavioral economics paradigms, the present research shows that exposure to the ligand causes differentiated behavioral effects in reciprocal punishments (Study 1) as well as rewards (Study 2), two types of behaviors that are frequently regarded as essential for the development and maintenance of cooperation.

## Introduction

Humans display substantial levels of cooperation among non-relatives. Although natural selection is conventionally assumed to favor selfishness and the maximization of own resources—if necessary—at the expense of others, human societies are organized around cooperative interactions (Nowak and Sigmund, 2005). Research from various disciplines suggests that reciprocity is the key to foster cooperative outcomes (Ostrom, 1990; Boyd and Richerson, 1992; Fehr and Gächter, 2000; Bowles and Gintis, 2004; Bear and Rand, 2016). In recent years, proximate mechanisms underlying the evolution of reciprocity and cooperation have received increasing research attention, implicating, for example, phylogenetically old cognitive mechanisms such as intuitive mental processing to relate to cooperation (Rand et al., 2012, 2014; Evans et al., 2015; Rand, 2016). At a more fundamental level, biochemical processes, for instance in trust and empathy, are less well understood and have primarily focused on the role of the neuropeptide oxytocin (Kosfeld et al., 2005; Bartz et al., 2010). Interestingly, this research has not yet successfully linked such bio-chemical processes to reciprocity (e.g., trustworthiness) and recent reviews even suggest that the cumulative evidence does not provide robust convergent evidence that human trust levels are reliably associated with oxytocin (Nave et al., 2015). Thus, whereas it is largely accepted that reciprocal behavior facilitates cooperation, the bio-chemical mechanisms that underlie and moderate this behavior remain largely unknown. The present research suggests chemosensation (i.e., chemical communication) to contribute to the explanation of human reciprocal impulses by combining research methods from neuroscience, physiology (Wallrabenstein et al., 2015), and experimental economics.

Whereas many animals use airborne chemicals to communicate, it remains a largely open and highly controversial research question whether humans may use chemosignals for social communication. Generally, since the classification as “microsomatic animals” (Turner, 1890), it seems commonly perceived that chemosensory perception plays only a minor role in humans. This view, however, seems at odds with the observation that the perfume industry is a multi-billion-dollar endeavor and the fact that odorants play a prominent role in human rituals from ancient times to modern day (Lübke and Pause, 2015). In animals, researchers often speak of “social odors” or pheromones, which are defined as chemicals that are released from one animal and evoke reproducible change in the behavior or hormonal system of another animal of the same species (Karlson and Luscher, 1959). Despite their prominence in several animal species, human chemosensory communication is discussed much more controversially (Turner, 1890; Wysocki and Preti, 2004; Wyatt, 2015). Therefore, researchers often avoid the term “pheromone” in favor of “chemosignal,” as no general consensus exists on what constitutes a (human) pheromone (Doty, 2010; Wyatt, 2014; Lübke and Pause, 2015). That said, scientists have identified multiple domains of human chemosensory communication, most prominently in human reproduction (Franzoi and Herzog, 1987; Sergeant et al., 2005; Havlícek et al., 2006; Mostafa et al., 2012; Lübke and Pause, 2015) as well as in harm avoidance (Mujica-Parodi et al., 2009; Zernecke et al., 2011; Lübke and Pause, 2015). Clearly, these domains are critical regarding the evolution of our species. But up to today, there is limited, if any, evidence that links another defining characteristic of human behavior—our ability to successfully cooperate with strangers—to chemosensory perception. Our research aims to fill that gap by investigating if a subtle application of an odorant that has been shown to affect chemosensory systems in humans (Wallrabenstein et al., 2015) may alter subsequent reciprocity in terms of costly reciprocal punishments and rewards in laboratory games.

So far, research efforts in the domain of chemosensory communication largely consists of three classes: the *sources* of human chemosignals (including bioactive molecules), the *perception* of human chemosignals, and the *functional significance* of chemosensory communication (i.e., their effects on hormonal levels, brain activity, or psychological variables related to emotion, cognition, and behavior). Research addressing the sources of chemosignal molecules in humans primarily focuses on the axillary as it hosts several glands whose secretion in the presence of resident bacteria contributes to a unique odorous “fingerprint” (Lübke and Pause, 2015). Furthermore, information about anxiety and stress is unconsciously transmitted via axillary sweat. Neuronal activation patterns suggest that areas linked to the processing of emotion and the regulation of empathy and attention were involved in these effects (Mujica-Parodi et al., 2009; Pause et al., 2009; Prehn-Kristensen et al., 2009). Beyond axillary sweat as a chemosignal, smelling of female tears that originate from sadness were shown to reduce activity in brain substrates of sexual arousal in males (Gelstein et al., 2011).

The perception of mammalian chemosignals is mainly achieved in the main olfactory epithel (MOE) and the vomeronasal organ (VNO). Partly, the controversy about humans' ability to rely on chemosignals may result from the knowledge that humans lack a functional VNO and have silenced most of the VNO receptor genes [i.e., only five genes (V1N1-5) are still functional; Wallrabenstein et al., 2015]. This does, however, not necessarily lead to the conclusion that chemosensory communication is entirely disabled (Witt and Hummel, 2006). For instance, surgical removal of the VNO in neonatal rabbits did not disable stereotypical nipple-search behavior; it occurred independently of the VNO via the main olfactory pathway (Distel and Hudson, 1985). Related results based on pigs lead to similar conclusions showing that chemosensory communication can be observed independently of the existence of a functional VNO. Importantly, the detection of “social odors” cannot only be accomplished by the VNO, because pheromone receptors (V1Rs and V2Rs) are also expressed in the main olfactory epithelium (Brennan and Zufall, 2006; Frasnelli et al., 2011; Wallrabenstein et al., 2015). Animal studies that are particularly relevant for our context include evidence on social cooperation in rodents. Research found that when all perceptual modalities were allowed for, rodents yielded a superior social cooperation performance (Avital et al., 2016).

In humans, derivatives of sex hormones may qualify as potential single molecules evoking physiological, hormonal, or behavioral responses in other humans. For example, androstene molecules have, among others, been linked to chemosensory communication due to their influencing of mood, physiological arousal, visual perception, and brain activity (Jacob et al., 2001; Savic and Berglund, 2010). Recently Hedione (HED), an ester and aroma compound with a jasmine-like smell created in chemical synthesis was identified as the first ligand activating one of the human VNO receptors (VN1R1). Using Ca-Imaging measurements it could be demonstrated that HED activates the recombinantly expressed human VN1R1 receptor, which is discussed as a putative pheromone receptor according to its structural homology to receptors detecting pheromones in mice (Boschat et al., 2002; Wallrabenstein et al., 2015). Second, using functional magnetic resonance imaging (fMRI), it was found that *in vivo* administration of HED exhibited a significantly enhanced activation in limbic areas (amygdala, hippocampus) and elicited a sex-differentiated response—being ten times larger in women—in a hypothalamic region that is associated with hormonal release in comparison to a common floral odor PEA (phenylethyl alcohol) (Wallrabenstein et al., 2015). These results are particularly relevant for the present research because HED incurred strikingly similar brain reaction compared to the sexual hormones AND and EST. Therefore, HED may be a powerful odorant as it acts *like* a human chemosignal, but does not require the exact identification of such.

Given these results on HED (Wallrabenstein et al., 2015), we used experimental economic games to capture potential effects on human behavior. The guiding hypothesis is that HED may be associated with elevated levels of negative reciprocity (i.e., punishments) as well as positive reciprocity (i.e., rewards). Both types of behavior have been linked to the evolution of cooperation and may therefore be a “useful” mechanism for social-chemical communications in humans.

## Materials and methods

Both reported experiments were conducted at the Cologne Laboratory for Economic Research, an economic laboratory with a capacity of 32 separate cubicles. Experiments were realized using the software z-Tree (Fischbacher, 2007) for experimentation and ORSEE (Greiner, 2015) for recruiting of participants. We deliberately opted to perform the experiments at the University of Cologne, where participants are highly unlikely to have been confronted with research around social-chemical communication before (e.g., through lectures, seminars), as this type of research has not been conducted at the University of Cologne's economic laboratory before. Therefore, the location made potential recognition of odorants next to impossible and hypothesis guessing of participants unlikely. Our main treatment manipulation was the exposure of experimental participants to the odorant Hedione. In Study 1, HED was contrasted against no odor (as was done in closely related research on reciprocity; Liljenquist et al., 2010), while in Study 2, we contrasted HED against a PEA control, due to its similarity to HED in terms of intensity and pleasantness (Wallrabenstein et al., 2015), to estimate a causal effect of Hedione on reciprocity in humans.

A total of 188 participants were recruited for the two cash-incentivized choice experiments. The computerized experiments were designed and analyzed closely following previous literature on reciprocity and punishment (Mussweiler and Ockenfels, 2013). Our approach involved a standard game of negative reciprocity (two-person simultaneous public goods game with a subsequent punishment phase; *n* = 60) and a standard game of positive reciprocity (two-person sequential trust game; *n* = 64 trustees and *n* = 64 trustors, who did not have a decision that involved reciprocity). Using a “between-subjects” design, participants were randomly assigned to sessions that were either scented with HED (experimental condition) or a control condition (Study 1, unscented control, Study 2, PEA scented room). All odorants were dissolved in ethanol (1:100) and applied on a cotton placemat approximately 15 min prior to participants' arrivals, allowing ethanol to evaporate. The odorants used included phenylethyl alcohol (PEA, from Aldrich, Steinheim Germany) as control in Study 2) and the V1N1 agonist Hedione (Firmenich, Meyrin, Switzerland, used in 5% solution in propylene glycol) as treatments.

Participants were randomly assigned to a seat in the laboratory that can accommodate 32 participants at the same time. All participants made decisions either about positive or negative reciprocity using the strategy method (i.e., conditional on the others' cooperation in either the public goods or the trust game). Subsequently, trust game participants (Study 2 on reciprocal rewards) received a post-experimental questionnaire to assess demographics as well as the variables used in the regression models (ratings of perception, valence of the odorants, etc.). During this questionnaire, participants received a test strip to smell the odorants and to rate perception (yes vs. no) and pleasantness on a scale ranging from 1 (very unpleasant) to 5 (very pleasant). After the trust game, but before the outcomes were announced, we also administered positive and negative affect scales to control for inflated mood incurred by either odorant. All instructions to the games are provided upon request. Following all ethical guidelines of the Cologne Laboratory for Economic Research, the studies did not need to be re-approved by the ethical review board and was performed under the approval of the laboratory.

To test the hypothesis that HED increases reciprocity in humans, we first examined whether it is related to reciprocal punishment. Individual willingness to punish non-cooperative others, even when this comes at a cost to the punisher, has been identified as an important driver of cooperation (Boyd and Richerson, 1992; Fehr and Gächter, 2002; Nowak, 2006). Research in many scientific disciplines shows that the presence of a punishment option is an effective institutional mechanism to promote cooperation (Fehr and Gächter, 2002; Mussweiler and Ockenfels, 2013; Feinberg et al., 2014). In Study 1, we therefore tested whether punishment decisions are affected by HED. We employed a standard game of negative reciprocity taken from previous research (Mussweiler and Ockenfels, 2013). Two participants always formed a group. At stage 1, participants had the chance to simultaneously transfer money to the other person, which was augmented by the researchers. The transfer of 1 monetary unit increased the payoff of the interaction partner by 2 monetary units. Hence, it was collectively optimal that both group members transfer their entire endowment to the other person, whereas selfishness dictates free-riding and implying a transfer of zero to the other player. Thus, if both players behave selfishly, both are worse off compared to cooperative behavior. At stage 2, participants received another 6 monetary units that could be used to reduce the others' payoff. Each monetary unit used for punishment would reduce the payoff of the other person by 3 units. Punishment decisions were measured by means of the so-called “strategy method,” meaning that each individual was asked, before the stage 1 decisions were disclosed, how many points she would assign as a punishment *conditional* on each potential transfer of the other person. This allows us to observe how punishment changes as the cooperation level changes, for all possible levels, and thus to get a complete picture of negative reciprocity. After all decisions were made, payoffs were realized and paid out in cash.

Following the experiment that tested the effect of HED on punishments in a game of negative reciprocity, we also tested the effect of exposure to HED in a game of positive reciprocity. First, the demonstration of such an effect addresses the robustness of our initial result in another important domain of reciprocal behavior. Second, we also gathered several control variables to account for potential alternative explanations. To test the hypothesis that HED increases positive reciprocity in humans, we used a trust game variant measuring trust and positive reciprocity taken from previous research (Mussweiler and Ockenfels, 2013) and selectively manipulated exposure to either HED or a control stimulus (PEA, phenylethyl alcohol) similar in intensity and valence, but which does not activate the V1NR1 and, more importantly, does not incur similar neurological responses like HED (Wallrabenstein et al., 2015). In stage 1, participants in the role of the trustor decided how much, if any, money send to an anonymous interaction partner (i.e., the trustee) present in the laboratory. Each unit sent was doubled by the researchers to secure efficiency gains of cooperation. Subsequently, without yet disclosing the decision from stage 1, participants in the role of the trustee were asked to decide how much to reciprocally reward the other player's cooperation level at stage 1 *conditional* on all possible amounts that this interaction partner could send (i.e., using again the “strategy method”). Here, we thus observe how the rewards change as the cooperation level changes, for all possible levels, and are thus able to get a complete picture of positive reciprocity.

## Results

### Negative reciprocity

**Figure 1:**
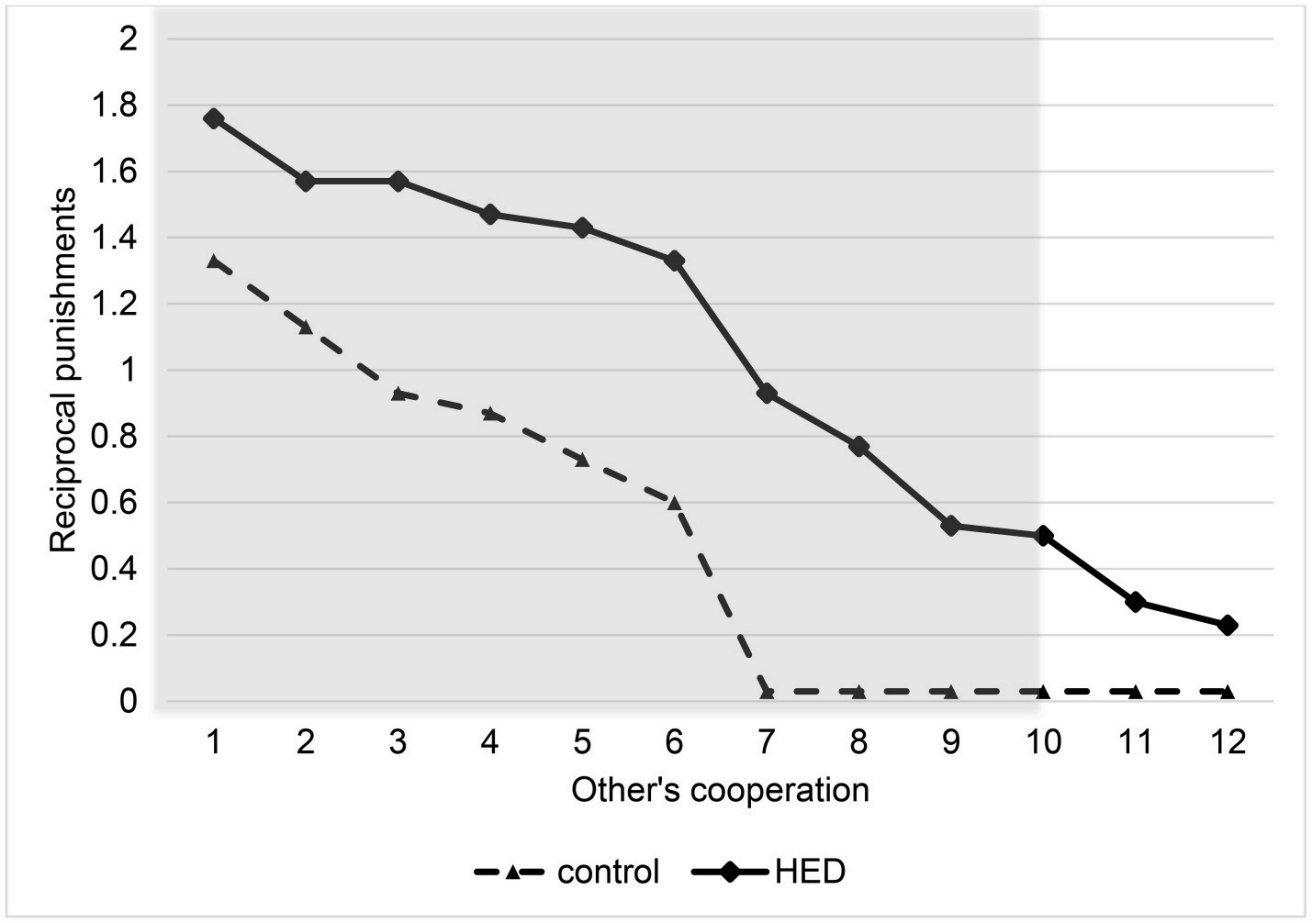
**Reciprocal punishments depending on other's cooperation level and exposure to HED, figure displays descriptive data (mean value assigned for punishment at various levels of other's cooperation) and shows a positive effect of HED on reciprocal punishments**. Gray area highlights area in which interaction effect (other's cooperation × HED) is particularly expected.

Random-effects panel regressions that account for potential censoring of the dependent variable (Tobit) were used to estimate the effect of HED on punishment behavior. Tobit regression had to be used instead of linear regression because behavior is restriced at both sides (i.e., it is impossible to punish less than zero and more than the endowment). Our results show a significant *others-cooperation-treatment* interaction (*P* < 0.05, obtained from regression results) in the domain of negative reciprocity, suggesting that the presence of HED led to increased punishment depending on the first order pro-social behavior of the interaction partners. Figure 1 depicts this interaction, showing that HED is associated with a stronger dependency of punishment on the other's willingness to cooperate. The gray area highlights the region in which the effects emerge. As expected, the effects emerge particularly at low levels of the other's cooperation. The regression is displayed in Table 1.

**Table 1:** **Regression results (Reciprocal punishments, Study 1)**.

	**Model 1**
Treatment (1 if HED)	4.2174
	(3.7788)
Other's cooperation	−1.0671^***^
	(0.1201)
Treatment × Other's	0.3481^***^
Cooperation	(0.1328)
Own cooperation	1.3918^***^
	(0.4963)
Treatment × Own cooperation	−0.4719
	(0.5925)
Constant	−10.9134^***^
	(3.4717)
Observations	780
Subjects	60

Coefficients obtained from random-effects Tobit regressions with standard errors in parentheses, statistical significance is indicated by

*** *p < 0.01*,

^**^p < 0.05, ^*^p < 0.1.

### Positive reciprocity

**Figure 2:**
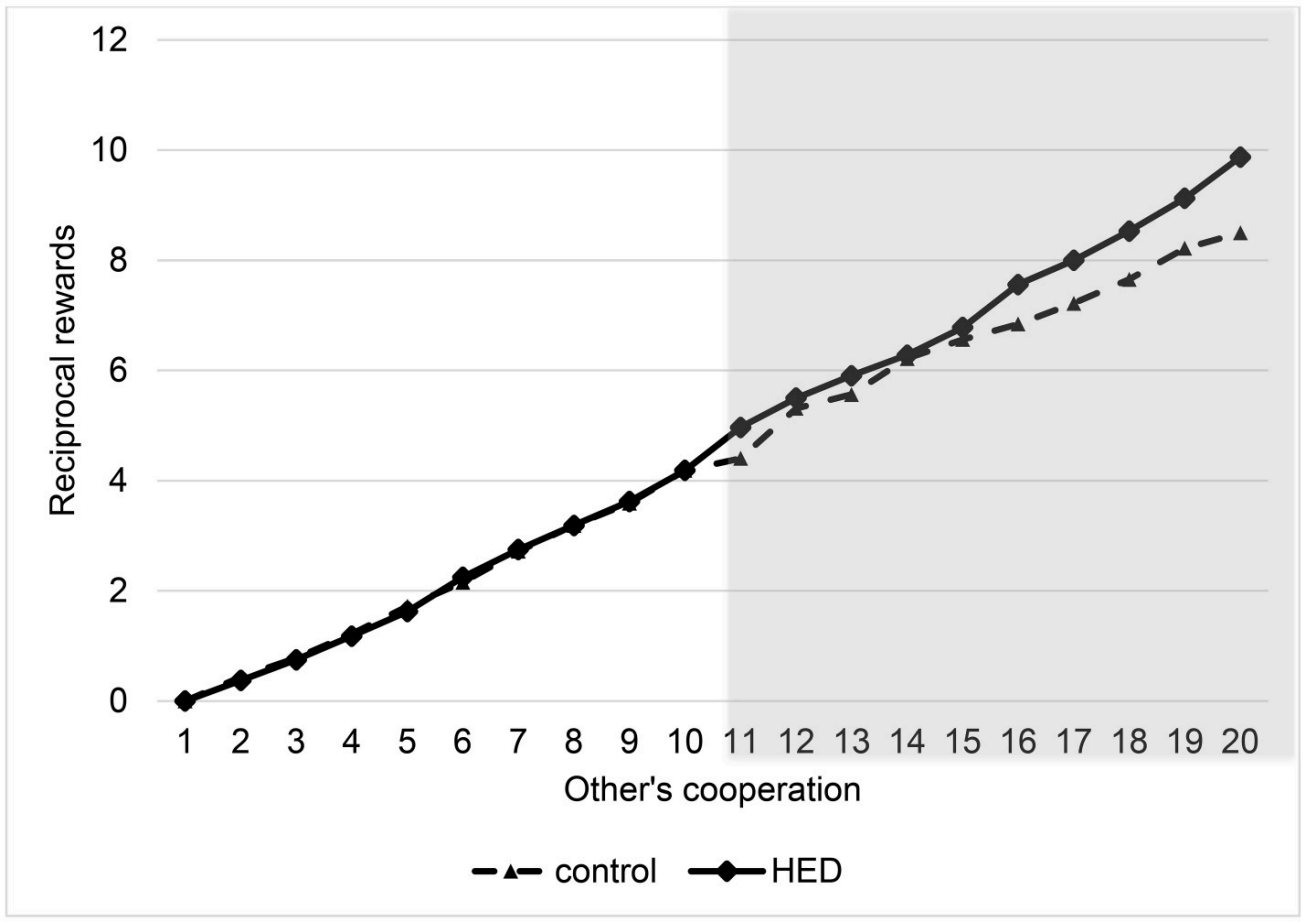
**Reciprocal rewards depending on other's cooperation and exposure to HED, figure displays descriptive data (mean value assigned for rewards at various levels of other's cooperation), and shows a positive effect of HED on reciprocal punishments**. Gray area highlights area in which interaction effect (other's cooperation × HED) is particularly expected.

To analyze the results, we again used random-effects panel regressions that account for potential censoring of the dependent variable (Tobit). Results show a significant *others-cooperation-treatment* interaction (*P* < 0.001, obtained from regression analysis), suggesting that the presence of HED led to more reciprocity; that is, a stronger reaction of the rewards to changes in the other's cooperation level. Figure 2 depicts this interaction. It shows that HED is associated with higher rewards only for high cooperation levels of the interaction partner. However, Figure 2 also indicates that there is no effect for small and medium amounts of sent money, probably because anything but large amounts may not necessarily signal “trust,” and so the reward effect for more mediocre levels of cooperation might be diluted. The gray area in Figure 2 highlights the region in which the effects emerge.

Next, several robustness tests were calculated to support the conclusion that HED is reliably associated with increased reciprocity (regressions are presented in Table 2). First, biological research pointed to gender-specific responses of pheromone-like substances, among them HED. Most importantly, it was previously shown that HED is perceived to be more intense by women. Also the HED-incurred brain response was found to be much stronger in women compared to men (Wallrabenstein et al., 2015). This finding fits into related research suggesting, for example, that androstenol, an odor frequently claimed responsible for human chemosensory communication, also results in gender-differentiated activation in the hypothalamus with effects being particularly found for females (Savic and Berglund, 2010). Hence, our first series of robustness checks (Models 2–3) attempt to capture potential gender-specific differences in the effect of HED on reciprocity. Simply controlling for gender yields a marginal statistical effect of gender (*P* = 0.056, obtained from regression analysis) and confirms the significant *others-cooperation-treatment* interaction effect (*P* < 0.001, obtained from regression analysis). A regression (Model 3) that includes a *gender-others-cooperation-treatment* three-way interaction to test the gender-differentiated effect showed a significant three-way interaction and rendered the *others-cooperation-treatment* two-way interaction insignificant. Thus, the results fit into a general pattern that highlights gender differences in the physiological response to the tested odorants (Wallrabenstein et al., 2015). As in previous research, our results are particularly driven by female experimental participants.

**Table 2:** **Regression results (Reciprocal rewards, Study 2)**.

	**Model 1**	**Model 2**	**Model 3**	**Model 4**	**Model 5**
Treatment (1 if HED)	−1.208	−0.855	−0.673	−1.583	−1.989
	(2.274)	(2.192)	(2.188)	(2.979)	(2.640)
Other's cooperation	0.686^***^	0.686^***^	0.772^***^	0.625^***^	0.685^***^
	(0.0210)	(0.0210)	(0.0338)	(0.0252)	(0.0210)
Treatment × Other's	0.142^***^	0.142^***^	0.0630	0.232^***^	0.142^***^
Cooperation	(0.0305)	(0.0305)	(0.0481)	(0.0349)	(0.0305)
Gender (1 if female)	–	4.157^*^	5.135^**^	–	–
		(2.173)	(2.203)		
Gender × Other's	–	–	−0.139^***^	–	–
Cooperation			(0.0426)		
Treatment × Other's	–	–	0.127^**^	–	–
Cooperation × gender			(0.0612)		
Perceived odorant (1 if	–	–	–	0.600	1.928
yes)				(3.223)	(2.456)
Rating of odorant	–	–	–	0.911	–
				(1.033)	
Positive affect	–	–	–	–	1.102
					(1.444)
Constant	−6.316^***^	−8.631^***^	−9.303^***^	−11.91^**^	−9.554^**^
	(1.642)	(2.037)	(2.030)	(5.415)	(4.154)
Observations	1,344	1,344	1,344	1,134	1,344
Subjects	64	64	64	54	64

Coefficients obtained from random-effects Tobit regressions with standard errors in parentheses, statistical significance is indicated by

*** *p < 0.01*,

** *p < 0.05*,

* *p < 0.1. n = 10 subjects did not answer the question “rating of odorant” in Model 4*.

Second, it is also plausible that differences in the ability to perceive the odorants, perceived valence of the odorants, or induced mood differences may contribute to the identified positive effect of HED on reciprocity. Therefore, a second class of robustness checks (Models 4–5) includes the general ability to perceive the odorants and their subjective hedonic ratings, as well as affect measures as statistical controls. As it is not directly possible for participants to consciously perceive HED or PEA when entering the laboratory, we gave participants test strips once they finished the experiment and asked them whether or not they perceive an odorant and, if yes, how pleasant they rate the odorant (see Methods section). These variables served as the relevant control variables. In addition, we administered positive and negative affect using the PANAS scale (Watson et al., 1988). This affect measure was collected after the decisions have been made, but before participants were informed about their payoffs. The *others-cooperation-treatment* interaction remains statistically significant when controlling for any of these control variables.

To sum up, HED significantly increased positive reciprocity in an experimental economic game and the effects cannot be solely attributed to perception and perceived valence or the odorant, as well as mood differences incurred by substance administration. As in previous research, the effects may be driven particularly by female participants. In sum, our studies provide initial evidence that exposure to HED may be associated with human reciprocal impulses in the domains of negative and positive reciprocity.

### Effects of hedione on trust, cooperation, and affect

Although not the primary interest of the present paper, we analyzed potential effects of HED on first-order behavior as well as affect ratings. We tested whether HED led to higher trust, cooperation, or different responses on the PANAS scale. However, HED did not have an effect on behavior in in stage 1 of the punishment game (*t*-test, *P* = 0.834) and behavior in stage 1 of the trust game (*t*-test, *P* = 0.651). This indicates that, while HED directly affects reciprocity, there seems to be no immediate or anticipatory effect on the kind of risky decisions in the first stages of our studies, which trigger the reciprocal responses. Neither did we observe direct effects of HED on ratings of positive (*t*-test, *P* = 0.896) or negative (*t*-test, *P* = 0.960) affect, which were measured in the study on positive reciprocity. These results seem in line with previous laboratory evidence that shows that reciprocal behavior (both in terms of rewards and punishments) is an immediate impulse necessary to trigger cooperative outcomes in the long term (Fehr and Gächter, 2002; Gürerk et al., 2006; Gächter et al., 2008).

## Discussion

Two experiments demonstrated that exposure to the chemically synthesized odorant Hedione affects reciprocal behavior of experimental participants in the domain of negative and positive reciprocity. Our research is the first to use an odorant known to be a ligand of a putative human pheromone receptor to explain reciprocal behavior in experimental economic games. Our research sheds new light on several existing lines of research.

First, our research informs theorizing about the origins of human cooperation. Various models on the evolution of cooperation suggest some kind of assortment where individuals correctly predict others' strategies may have been involved in the evolution of cooperation. For instance, “green-beard theories” (Gächter et al., 2008) suggests that humans may be able to perceive and, more importantly, discriminate their behavior based on individual characteristics, like a hypothetical green beard that signals altruism. Probably most importantly, tremendous research effort has been undertaken to derive such information from the human face. Although there is a broad consensus about what constitutes a trustworthy face (Engell et al., 2007; Oosterhof and Todorov, 2008; Todorov et al., 2009; Dawkins, 2016), the evidence does not suggest that humans are able to reliably and robustly predict actual trust behavior or trustworthiness in experimental games (Ockenfels and Selten, 2000; Willis and Todorov, 2006; Efferson and Vogt, 2013; Rule et al., 2013). Our research critically informs this research line as chemosensory communication, the probably oldest form of communication between mammals, may be an alternative route to secure cooperation. If, indeed, humans use chemosignals to cooperate, it would de-emphasize the need for selective assorting.

Second, our results contribute to effects of chemosensory communication that have been previously observed. For instance, research suggests that men prefer female body odors when these were sampled from the axillary on women's most fertile days (Havlícek et al., 2006) and that generosity of men toward women is associated with the female ovulatory cycle (Miller et al., 2007). Potentially, our results that link olfactory perception and reciprocity can motivate further integration of these two lines of research by providing first support that behavioral effects can be found after chemosensory stimulation.

Third, our research informs existing work in social psychology that addresses the effects of smells on reciprocity. Research shows that human trustworthiness is associated to the presence of clean smells in the laboratory (Liljenquist et al., 2010). While this research builds primarily on an “embodied cognition” and a “conceptual metaphor” framework, our research adds a potentially alternative explanation for the observed effects. Single molecule might already invoke changes in behavior, as is the case for Hedione. Therefore, future research could disentangle these different explanatory paths that link the effects of odorants to reciprocal behavior to observe similar mechanisms as were found for psychological variables such as harm avoidance (Mujica-Parodi et al., 2009; Prehn-Kristensen et al., 2009; Zernecke et al., 2011).

Finally, it would be interesting to identify the natural ligand of VN1R1 in the human body, which may give some hints of the behavioral situation in which it may be used to affect reciprocity of our neighbors. Existing research on natural ligands has delivered interesting insights with respect to lactating mothers and neonates (Doucet et al., 2009), suggesting that secretion of areolar glands of non-related women elicits unconditional responses in 3-day old babies. Natural ligands involved in reciprocity may be much harder to identify, as reciprocity is heavily influenced by cultural factors as well.

Concluding, our results provide initial support for the role of human olfactory perception in regards to social behavior. Across two studies, we found support for our hypothesis that exposure to the chemically synthesized odorant Hedione, a substance found to incur interesting physiological reactions (i.e., it is an activator of the putative human pheromone VN1R1 receptor, neurological response), positively affects reciprocal behavior in economic games. Our results open up many questions on how humans may use social-chemical communication and a combination of physiological and experimental economic tools may serve as a suitable combination to test behavioral effects of “social odors.” Therefore, the results also augment the methodological toolbox for research in this domain, which has so far largely neglected paradigms adopted from behavioral economics to study human social behavior.

## Ethics statement

This study was carried out in accordance with the recommendations of the Cologne Laboratory for Economic Research with written informed consent from all subjects. All subjects gave written informed consent in accordance with the Declaration of Helsinki. The protocol was approved as the study was conducted under the general guidelines of the economic laboratory and did not require additional approval.

## Author contributions

SB, HH, and AO conceived the research and designed the experiments, revised the manuscript, and approved the final version and are accountable for the content of the manuscript. SB performed the experiments, analyzed the data with support of AO, and drafted the manuscript.

## Funding

SB gratefully acknowledges funding by the German Research Foundation (LO1826 1/1). SB and AO gratefully acknowledge funding by the German Research Foundation (FOR1371). The funding agency played no role in designing the research questions was not involved in discussion of the results prior to submission.

### Conflict of interest statement

The authors declare that the research was conducted in the absence of any commercial or financial relationships that could be construed as a potential conflict of interest.
